# Effects of *Curcuma longa *(turmeric) on postprandial plasma glucose and insulin in healthy subjects

**DOI:** 10.1186/1475-2891-9-43

**Published:** 2010-10-12

**Authors:** Jennie Wickenberg, Sandra Lindstedt Ingemansson, Joanna Hlebowicz

**Affiliations:** 1 Department of Medicine (JW, JH), Skåne University Hospital, Malmö, Sweden; 2 Department of Cardiothoracic Surgery (SL), Skåne University Hospital, Lund, Lund University, Sweden

## Abstract

**Background:**

Previous animal studies have shown that *Curcuma (C.) longa *lowers plasma glucose. *C. longa *may thus be a promising ingredient in functional foods aimed at preventing type 2 diabetes. The purpose of the study is to study the effect of *C. longa *on postprandial plasma glucose, insulin levels and glycemic index (GI) in healthy subjects.

**Methods:**

Fourteen healthy subjects were assessed in a crossover trial. A standard 75 g oral glucose tolerance test (OGTT) was administered together with capsules containing a placebo or *C. longa*. Finger-prick capillary and venous blood samples were collected before, and 15, 30, 45, 60, 90, and 120 min after the start of the OGTT to measure the glucose and insulin levels, respectively.

**Results:**

The ingestion of 6 g *C. longa *had no significant effect on the glucose response. The change in insulin was significantly higher 30 min (*P *= 0.03) and 60 min (*P *= 0.041) after the OGTT including *C. longa*. The insulin AUCs were also significantly higher after the ingestion of *C. longa*, 15 (*P *= 0.048), 30 (*P *= 0.035), 90 (*P *= 0.03), and 120 (*P *= 0.02) minutes after the OGTT.

**Conclusions:**

The ingestion of 6 g *C. longa *increased postprandial serum insulin levels, but did not seem to affect plasma glucose levels or GI, in healthy subjects. The results indicate that *C. longa *may have an effect on insulin secretion.

**Trial registration number:**

NCT01029327

## Background

*C. longa *(turmeric) is a tropical plant that is cultivated extensively in Asia, India, China, and other countries with a suitable climate. *C. longa*, is a perennial herb, and a member of the ginger family. It can grow up to 1 m high, and has oblong, tufted leaves. The yellow spice is made from the rhizomes (roots), which are boiled, dried, and then ground [1,2] The active component in turmeric is curcumin, which may constitute 2 to 8% of the spice. Curcumin is a non-water-soluble polyphenol that can be derived from *C. longa *by ethanol extraction [2]. *C. longa *has traditionally been used as a coloring agent in Asian cuisine, as well as in cheese, butter, yogurt, and other kinds of food [3].

*C. longa *is used for several purposes apart from flavoring and coloring food. Numerous studies have shown that curcumin has antioxidant and anti-inflammatory properties [4]. Recent studies have also indicated that curcumin affects cellular enzymes, and angiogenesis [5,6]. Although curcumin has been used throughout history, especially in India and Asia, the first study on curcumin and its dose-limiting toxicity was not published until 2001, when it was reported that amounts of up to 8 g, administered per day for three months, were not toxic to humans [7]. A long-term study on healthy subject revealed no changes in fasting plasma glucose or lipid levels when 2.8 g turmeric was given to the subjects daily for four weeks [8]. Diabetic rats given curcumin showed a significant reduction in renal dysfunction and oxidative stress [9], which may indicate that curcumin has a protective role against diabetic nephropathy.

Changes in lifestyle, such as increased energy intake and decreased physical activity, are causing overweight and obesity, leading to an epidemical increase in type 2 diabetes. It is well known that both obesity and type 2 diabetes increase inflammatory responses and cause metabolic disorders [10]. Low glycemic index (GI) and low glycemic load diets are associated with a reduced risk of type 2 diabetes, which is comparable to the risk reduction observed with a high intake of dietary fiber and whole-grain products [11]. Dietary intervention is thus very important in all stages of diabetes, and the identification of dietary components that can reduce the risk of developing diabetes, or complications associated with diabetes, would be valuable. The effects of *C. longa *on plasma glucose and insulin levels have not been studied previously in humans. This study was therefore designed to determine whether the ingestion of 6 g of *C. longa *in a single meal lowered postprandial plasma glucose and insulin levels in healthy subjects.

## Methods

Fourteen healthy subjects [seven males, seven females; (mean ± SD) age: 29 ± 1 y (range: 25 - 38 y); body mass index: 23.9 ± 2.7 kg/m^2 ^(range: 20.1 - 31.5 kg/m^2^)] were included in this crossover study. All subjects were recruited from the population of southern Sweden. Those who had a history of thyroid disorders or diabetes mellitus were excluded. The fasting plasma glucose concentration of each subject was checked on the day of the examination to ensure that it was normal (≤ 7.0 mmol/L). Three subjects were smokers and three were snuff users. The subjects were examined between eight and 10 a.m. after a 12-h fast.

Capsules containing 560 mg lactose (Apoteket, Produktion & Laboratorier, Gothenburg, Sweden) (placebo), or 170 mg lactose together with 400 mg *C. longa *(Svampbutiken, Mediapoint AB, Västerås, Sweden), were prepared in advance by the Malmö University Hospital Pharmacy. Although both kinds of capsules appeared identical, it is possible that some of the participants could discern differences between them. The reference OGTT consisted of 75 g/250 mL of a standard OGTT, ingested after swallowing 15 placebo capsules. The test OGTT consisted of the same amount of OGTT solution after taking 15 capsules containing the *C. longa*. The subjects were given 250 mL of water to swallow the capsules, and were asked to take them within five min. Extra lactose was added to the test OGTT (5.85 g) so that the amounts of lactose in both the reference and test OGTT, including the capsules, were the same. The tests were performed in random order at intervals of 1 week. Randomization was performed using a table of random numbers.

Finger-prick capillary blood samples were taken for glucose measurements, and venous blood for insulin measurements, before, and 15, 30, 45, 60, 90, and 120 min after the consumption of the meals. Plasma glucose concentrations were measured with the HemoCue Glucose system (HemoCue AB, Ängelholm, Sweden), which converts blood glucose to plasma equivalent glucose concentrations by multiplying by a constant factor of 1.11 [12]. The precision of the HemoCue Glucose system is/was better than 0.3 SD from 0 mmol/L to 22.2 mmol/L. Insulin concentrations were measured using an immunoassay with an alkaline phosphatase conjugate (Access Ultrasensitive Insulin, Beckman-Coulter AB, Bromma, Sweden). The sensitivity of the insulin immunoassay is/was 0.03 mUnit/L (mU/L), and the intra-assay coefficient of variation is/was below 10% in the range 0.03 mU min/L to 300 mU/L.

All subjects gave their written informed consent. The study was approved by the Ethics Committee of Lund University, and performed according to the Helsinki Declaration.

The incremental areas under the curves (AUCs) were measured for plasma glucose, and serum insulin in each subject (GraphPad Prism version 3.0; GraphPad Software, San Diego, CA, USA). The AUC was calculated above baseline. The GI and insulinemic index (GII) were expressed as the increase in area under the glucose curve following the test OGTT as a percentage of the same participant's response after the reference OGTT. The 0-90 min and 0-120 min AUCs were used to calculate the GI and GII [13,14]. All statistical calculations were performed using SPSS for Windows software (version 14.0, 2005). Differences in the plasma glucose levels, insulin levels, and glycemic index were evaluated with the Wilcoxon's *t*. Values of *P *< 0.05 were considered statistically significant. This study, employing fourteen healthy subjects, had an 80% power to detect a 20% change in GI at a level of *P *< 0.05.

## Results

### Postprandial plasma glucose response

**Figure 1:**
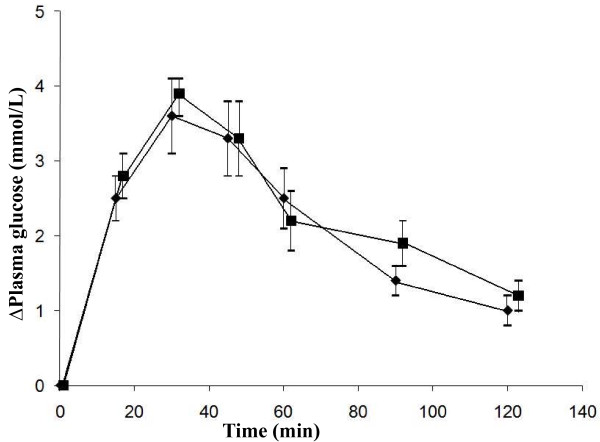
**The mean (± SEM) incremental plasma glucose concentration in fourteen healthy subjects after the OGTT with placebo capsules (reference) (black diamond) or *C. longa *capsules (black square)**. No statistically significant differences were found between the mean plasma glucose concentrations when evaluated with the Wilcoxon signed rank sum test.

The mean fasting plasma glucose level before the reference OGTT was 5.75 ± 0.13 mmol/L and was not statistically significantly different from that before ingestion of the test OGTT containing *C. longa *(5.76 ± 0.10 mmol/L). No significant differences were seen in the glucose response at different times, or in the differences between the areas under the postprandial glucose curves between the reference and test OGTT (Figure 1, Table 1).

**Table 1:** Postprandial plasma glucose area under the curve (AUC) and glycemic index (GI) in healthy subjects after the ingestion OGTT with a placebo (Reference) or the OGTT with 6 g *C. longa*.

	Glucose AUC (mU min/L)		GI (%)	
**Time**	**Reference**	***C. longa***	**Reference**	***C. longa***

0-90 min	216.5 ± 27.4	229.6 ± 23.8	100	137.9 ± 24.8
0-120 min	253.9 ± 32.7	275.6 ± 28.3	100	135.0 ± 20.7

All values are means ± SEM, *n *= 14. No significant differences were found between postprandial plasma glucose AUCs or GIs when evaluated with the Wilcoxon signed rank sum test. U = Unit

### Postprandial insulin response

**Figure 2:**
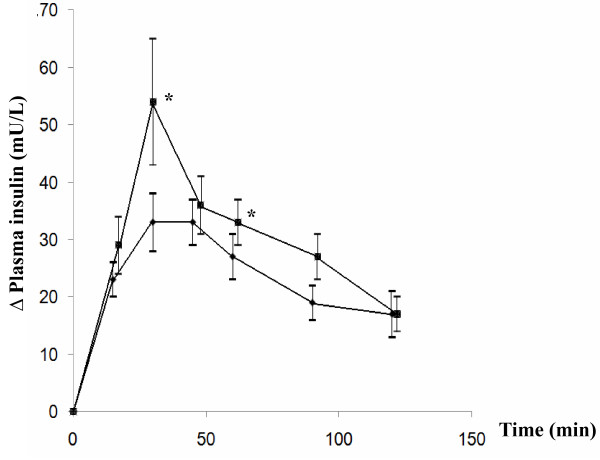
**The mean (± SEM) incremental serum insulin concentration in fourteen healthy subjects after the OGTT with placebo capsules (reference) (black diamond) or *C. longa*capsules (black square)**. * = Significant difference between the responses to the reference and test OGTT when evaluated with the Wilcoxon signed rank sum test, *P *< 0.05. U = Unit

Ingestion of 6 g *C. longa *resulted in a significantly higher serum insulin response 30 min (*P *= 0.048) and 60 min *(P *= 0.033) after the OGTT, compared with the reference OGTT. The change in insulin level (Δ insulin) was also significantly different from that seen after the reference meal 30 min (*P *= 0.03) and 60 min (*P *= 0.041) postprandially (Figure 2). The ingestion of 6 g *C. longa *also resulted in a significantly higher AUC 15 min (*P *= 0.048), 30 min (*P *= 0.035), 90 min (*P *= 0.03) and 120 min (*P *= 0.02) after the test OGTT than the reference OGTT (Table 2).

**Table 2:** Postprandial serum insulin area under the curve (AUC) in healthy subjects after the ingestion of OGTT with a placebo capsules (Reference) or the OGTT with *C. longa *capsules.

	Insulin AUC (mU min/L)	
**Time**	**Reference**	***C. longa***

0-15 min	169.2 ± 19.8	217.5 ± 37.7*
0-30 min	584.3 ± 61.2	838.1 ± 152.7*
0-45 min	1076.5 ± 111.4	1509.9 ± 252.2
0-60 min	1521.7 ± 147.3	2021.0 ± 285.9
0-90 min	2210.3 ± 215.5	2908.3 ± 353.0*
0-120 min	2749.5 ± 283.7	3570.9 ± 410.7*

All values are means ± SEM, *n *= 14. Significant differences in serum insulin AUCs were evaluated with the Wilcoxon signed rank sum test. * = Significant difference between the responses to the OGTT with and without *C. longa*, *P *< 0.05.

### The glycemic and insulinemic index

No significant differences were seen in GI between the reference and test OGTT (Table 1). The ingestion of 6 g *C. longa *resulted in a significantly higher GII 90 min after the OGTT (*P *= 0.024), while no significant difference was seen in GII 120 min after the OGTT (Table 3).

**Table 3:** Postprandial insulinemic index (GII) in healthy subjects after the ingestion of OGTT with a placebo capsules (Reference) or the OGTT with *C. longa *capsules.

	GI (%)	
**Time**	**Reference**	***C. longa***

0-90 min	100	136.4 ± 13.8*
0-120 min	100	130.0 ± 16.3

All values are means ± SEM, *n *= 14. Significant differences in GIIs were evaluated with the Wilcoxon signed rank sum test. * = Significant difference between the responses to the OGTT with and without *C. longa*, *P *< 0.05.

## Discussion

The aim of this study was to elucidate the effect of *C. longa *on postprandial glucose and insulin levels in healthy human subjects. The results show that the ingestion of 6 g *C. longa *increased postprandial serum insulin concentration without affecting plasma glucose in healthy subjects. The effect of *C. longa *on postprandial plasma glucose and insulin levels has not been studied previously in humans. Curcumin is the active component of *C. longa*, comprising about 2-8% of the spice, and has been found to have potent antioxidant, anti-inflammatory, and anti-carcinogenic properties [5,6,11]. A decrease in blood glucose and glycosylated hemoglobin levels has been observed when 0.08 g curcumin/kg body weight or 1 g *C. longa */kg body weight was administered to diabetic rats daily for three weeks [3]. It was also shown in the same study that curcumin and *C. longa *reduced oxidative stress. Overall, the study showed that curcumin was more effective than *C. longa *in mitigating the changes typical in diabetes mellitus.

In a similar study on diabetic rats given curcumin for two weeks (15 mg/kg and 30 mg/kg), a decrease in renal dysfunction was observed (measured as reductions in creatinine and urea clearance), proteinuria, together with a decrease in oxidative stress (measured as decreased activities of the key anti-oxidant enzymes) [9]. This indicates that curcumin may offer protection against diabetic nephropathy. In an *in vitro *study in which the effect of curcumin on human endothelial cells was investigated, the cells were found to have an enhanced cellular resistance to oxidative damage [15]. Obese mice with diabetes showed a significant improvement in glycemic control and insulin sensitivity when treated with 3% dietary curcumin for five weeks [10]. The improvement in glycemic status was associated with anti-inflammatory effects. Curcumin led to a decrease in NF-kB (nuclear factor kappa-light-chain-enhancer of activated B cells) activity in liver tissue and a decrease in macrophage infiltration, which can explain the antidiabetogenic effects seen in adipose tissue following the ingestion of curcumin. The study also showed that a fat-derived hormone, adiponectin, increased dramatically. Decreased adiponectin has been correlated with the development of insulin resistance [16]. Recent studies show that adiponectin also has anti-inflammatory effects on endothelial cells, thus preventing atherosclerosis. Generalized inflammation and atherosclerosis are two processes in which macrophages plays a major roll [17,18]. Reduction of the infiltration of macrophages into adipose tissue may be explained by the effect of curcumin on adiponectin [10].

The animal studies described above were long-term studies, in which various changes in the animals were measured after a few weeks or months of administration of curcumin or *C. longa*. In the present study, we measured the short-terms effect on plasma glucose and insulin levels in healthy humans. The results of our study show that the ingestion of 6 g *C. longa *increased postprandial serum insulin concentrations without significantly affecting plasma glucose levels, indicating that *C. longa *may have an effect on insulin secretion. The increased insulin response resulting from *C. longa *is probably due to the stimulation of beta-cell function by curcumin. This is consistent with the results of a previous *in vitro *study showing that curcumin enhances insulin release in pancreatic beta-cells [19]. In healthy subjects, glucose levels are strictly regulated, and it is difficult to measure differences in plasma glucose levels. This could explain why we did not see any significant differences in plasma glucose levels.

Further investigation of the effect of *C. longa *on glucose and insulin levels in patients with type 2 diabetes mellitus is needed. *C. longa *may be an ingredient in functional foods aimed at preventing type 2 diabetes, but the taste of *C. longa *and the amount required pose problems. It is impossible to eat 6 g of *C. longa *in food because of its taste. Another issue is the bioavailability of curcumin. It has been shown in recent studies that curcumin has poor bioavailability because of its poor absorption and rapid metabolism. Several studies have indicated that the amount of curcumin in the serum after an intake of 4-8 g is only 0.4-3.6 μM [20]. *C. Longa *with its curcumin is probably a very healthy spice in the long term, but unfortunately it has a very poor bioavailability and it cannot be ingested on its own.

## Conclusions

The present study shows that the ingestion of *C. longa *increased postprandial serum insulin levels, but did not affect plasma glucose levels or GI in healthy subjects. The results indicate that *C. longa *may have an effect on insulin secretion.

## Competing interests

The authors declare that they have no competing interests.

## Authors' contributions

The authors' contributions were as follows: JW and JH responsible to the design of the study; JW was responsible for recruiting the subjects and carried out the practical aspects of the study. JW and JH conducted the statistical calculations and created the graphs. JW and JH wrote the first draft of the manuscript and SL made critical revisions of the manuscript. All the authors read and approved the final manuscript. None of the authors had any personal or financial conflicts of interest.
